# An Implantable Neural Sensing Microsystem with Fiber-Optic Data Transmission and Power Delivery

**DOI:** 10.3390/s130506014

**Published:** 2013-05-10

**Authors:** Sunmee Park, David A. Borton, Mingyu Kang, Arto V. Nurmikko, Yoon-Kyu Song

**Affiliations:** 1 School of Engineering, Brown University, Providence, RI 02912, USA; E-Mails: sunmee_park@brown.edu (S.P); borton.brown@gmail.com (D.A.B.); arto_nurmikko@brown.edu (A.V.N.); 2 Department of Transdisciplinary Studies, Seoul National University, Seoul 151-744, Korea; E-Mail: whitymk@snu.ac.kr; 3 Advanced Institutes of Convergence Technology, Suwon 443-270, Korea

**Keywords:** brain machine interface, neural probe array, neuromotor prosthesis, optical telemetry, photovoltaic device

## Abstract

We have developed a prototype cortical neural sensing microsystem for brain implantable neuroengineering applications. Its key feature is that both the transmission of broadband, multichannel neural data and power required for the embedded microelectronics are provided by optical fiber access. The fiber-optic system is aimed at enabling neural recording from rodents and primates by converting cortical signals to a digital stream of infrared light pulses. In the full microsystem whose performance is summarized in this paper, an analog-to-digital converter and a low power digital controller IC have been integrated with a low threshold, semiconductor laser to extract the digitized neural signals optically from the implantable unit. The microsystem also acquires electrical power and synchronization clocks via optical fibers from an external laser by using a highly efficient photovoltaic cell on board. The implantable unit employs a flexible polymer substrate to integrate analog and digital microelectronics and on-chip optoelectronic components, while adapting to the anatomical and physiological constraints of the environment. A low power analog CMOS chip, which includes preamplifier and multiplexing circuitry, is directly flip-chip bonded to the microelectrode array to form the cortical neurosensor device.

## 1. Introduction

Neurotechnology has the potential to restore or replace lost functions in neurologically impaired humans. The field has already produced impressive developments, including cochlear implants and deep brain stimulators, which are in widespread clinical use today. Movement loss due to a neurological disability is devastating, and has produced a large patient population for whom there are limited available therapeutic options. A neuromotor prosthesis (NMP) is a neurotechnology that may restore lost movements in paralyzed patients; the development of a novel implantable sensor technology for cortical NMP is the subject of this paper.

Permanently implanted cortical microelectrodes measure signals from regions of the region of the brain that are directly related to intended movements [1–3]. For example, chronically implanted, multielectrode arrays have allowed long term (>1 year) exploration of reach and grasp representation in the motor cortex of rhesus macaques [4]. In conjunction with newly developed decoding techniques using probabilistic analysis [3,5], good correlation has been achieved for the arm movement of a monkey between the signals recorded directly from the brain and the real physical action by the animal [6]. A recent culmination of research into NMP development has been human clinical trials where intention-driven neuronal ensemble activity has been converted into a control signal that enables a tetraplegic patient to perform useful tasks [7–9].

Many approaches for chronic multichannel cortical recording involve a passive (*i.e.*, unpowered) implanted neural probe with percutaneous cabling to electronic processors outside the body that is often bulky and fragile [10–12]. The integration of a significant portion or all of a neural interface system onto an implantable platform is highly desirable for future portable and wearable prosthetics, including on-board auxiliary telemetry to transmit signals to internal or external remote processors and other assistive technologies. Implantable cortical neural interface microsystems present multifaceted technical challenges including the development and integration of ultralow-power microelectronic chips to the neuroprobe recording platform, approaches to on-board data telemetry, and the means to deliver power to the active components. Finally, encapsulation and biocompatibility of such an electrically active multielement implant are of fundamental importance for future human applications, in conjunction with surgical and other clinical considerations.

Early progress has been reported in the first steps along the development path towards the goal of fully implantable neural interface microsystems. For example, prototypes of integrated sensors, amplifiers, and wireless transmission have been reported [13–19]. In our own work, we have fully integrated a CMOS chip composed of low-noise analog preamplifiers and multiplexers onto the microelectrode array [20,21].

In this paper we describe a complete NMP microsystem developed in our laboratory and review its design and *in-vivo* and benchtop performances. Here we place particular emphasis on the optoelectronics components, which have enabled forward and backward telemetry as well as power delivery to the system through optical fibers. Some commercial neural recording systems (e.g., Cerebus™ from Blackrock Microsystems LLC, Salt Lake City, UT, USA and RZ2 from TDT Co. Alachua, FL, USA) have employed IR data link for the high fidelity broadband data transmission in an electrically isolated manner with light, flexible, and robust optical fibers. However, those are bulky head-mount systems that consume more than an order of magnitude larger power than an implantable microsystem presented here (not to mention the extreme contrast in the form factors). Also, we have previously developed a microcrystal photovoltaic device for converting infrared light to electrical energy to electrically stimulate the nerves and muscles to restore the motor functions [22,23]. Since the energy converter is capable of delivering enough power to stimulate muscles, it could be used to provide reasonable amount of power to the implantable low power microsystem. Thus, we have utilized an optical power supply based on such a photovoltaic microcrystal device in tandem cell configuration, not only for delivering power to drive active components in the microsystem, but also for providing clock signals to synchronize all the timing sensitive electronics in the system. We have employed biocompatible silica/polymer optical fibers carefully aligned to the micro laser (VCSEL) and optical power supply devices that are chip-level integrated on board, which ensures high efficiency delivery of infrared light with the on-board optoelectronic devices in the extremely small form factor. The fiber optic data transmission and power delivery enables broadband data transmission, efficient and safe power transfer, and complete electrical isolation for the implantable microsystem, which could add some flexibility in designing the future fully implantable neural prosthetic microsystems, as we call it “fiber to the brain”.

The full microsystem design is based on a specific device layout geometry and architecture that is aimed primarily at non-human primates and is composed of analog and digital microelectronic components as well as infrared optoelectronic devices. The microsystem has a soft and flexible encapsulation and its design principles emphasize scalability to adapt to an increasingly larger amount of neural information transmitted from the cortex to the external devices.

Finally, the entire microsystem with fiber-optic data transmission and power delivery has been tested in a bench top as well as *in vivo* animal model using an anesthetized rat, demonstrating a practical utility of our microsystem architecture for advanced neural prosthetic applications.

## 2. Design Rationale and Approach for an Implantable Neural Sensing Microsystem

The design of a fully implantable microsystem for cortical recording that incorporates wide bandwidth transcutaneous telemetry presents a multifaceted set of technological challenges and biomedical constraints. For example, at a fundamental level, the key constraint in an active microsystem concerns the heat imparted by active elements to the adjacent brain and other surrounding tissue. At the practical operational level, another important consideration is the surgical and clinical compatibility of the implant, including its reliability and the ability to monitor the performance of the system in real-time.

**Figure 1.**
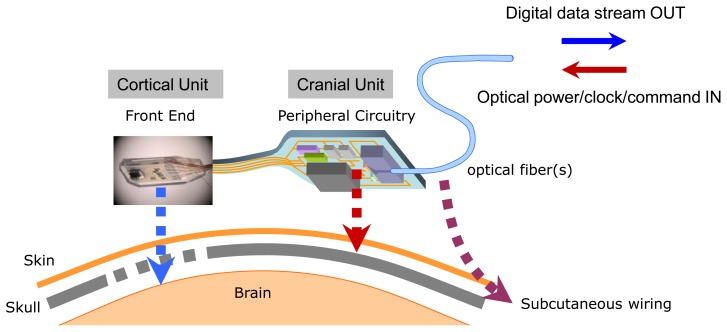
Schematic of the implantable microsystem architecture and anatomical placement strategy.

From a purely engineering viewpoint, the concept of using a cortical microelectrode array as a platform onto which *all* the microelectronic and telemetric components are directly integrated as a single monolithic cortical implant module might at first appear to represent an attractive approach. However, by weighing a number of neurophysiological, biomedical, telemetric, and surgical considerations, we have chosen to pursue a spatially distributed microsystem architecture shown schematically in Figure 1. The layout in the figure reflects our “dual-panel” microsystem design concept, composed of two electrically interconnected islands which are landscaped on a common flexible polymer (polyimide or liquid crystal polymer) substrate. The dual-panel system, detailed in this paper, is composed of a “front end” which is directly implanted into the cortex. This front panel is flexibly connected to the back panel which threads through a sealable “burr” hole in the skull and resides between the skull and the skin. The front end houses the cortical microelectrode array, which is directly flip-chip bonded onto a low power analog CMOS chip [20]. The custom designed and fabricated CMOS IC includes preamplifiers and multiplexing circuitry whose performances have already been reported elsewhere [20].

**Figure 2.**
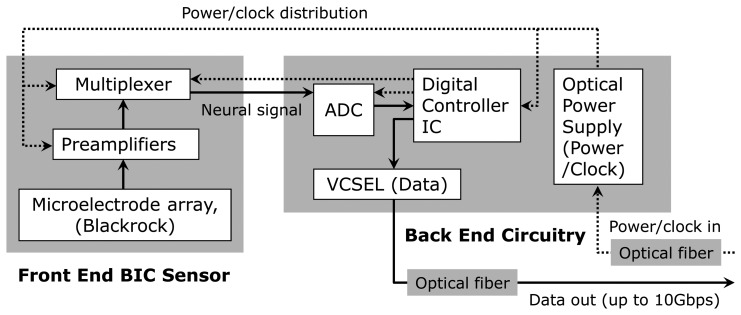
Block diagram for the implantable microsystem detailing the key micro- and optoelectronic on-chip circuit elements.

Figure 2 shows a block diagram of the full dual-panel system layout with its microelectronic and optoelectronic components in functional groups. The multiplexed analog neural signals are routed from the front panel to the peripheral circuits of the second panel (“back end”) of the implant. The back end circuitry integrates a low power analog-to-digital converter (ADC), a digital control-and-command chip, a semiconductor microcrystal photovoltaic energy converter (labeled as the “optical power supply” in Figure 2), and an infrared (IR) data link employing an infrared (IR) vertical cavity surface emitting semiconductor laser (VCSEL) with very low threshold current that has been successfully used for neural data transmission through skin [24]. In the microsystem described in the work, a pair of optical fibers directly connect the optical power supply and VCSEL to the external system to receive power/clock signals and transmit digitized neural data.

## 3. Microsystem Fabrication

In this section we describe the fabrication of the neural microsystem by focusing on the various subsystem components and their integration. The integration of a microsystem can be divided into two categories depending upon the requirements of alignment precision: chip-to-chip integration and component integration. “Chip-to-chip” integration includes the attachment of a 4 × 4 microelectrode array to a CMOS amplifier IC in our front end. Alignment of optoelectronic microcrystal chips (such as semiconductor lasers and photovoltaic cells) to optical subassemblies falls into this category as well. Component integration involves the placement of discrete parts and assemblies onto the substrate with relaxed alignment tolerance. Here we first briefly review the CMOS analog IC-driven front end, which is integrated into the microelectrode array, and show its performance through in-vivo cortical recording from rats under acute conditions described in Section 3.1. This is followed by an outline of the flexible polymer substrate design and processing in Section 3.2. We then proceed to discuss the peripheral circuitry (“back end”) and the implementation of its integrated components in Section 3.3. Some commentary on the important subject of encapsulation is given in Section 3.4, followed by a demonstration of the operation of the full microsystem in Section 3.5.

### 3.1. CMOS Microelectronic Chip and Front End Integration

We review here the steps of flip-chip integration and operation of the “front end” cortical implant chip-scale unit, which integrates a silicon-based multielectrode array with a high performance silicon integrated circuit. In the overall architecture of our CMOS amplifier array, each preamplifier possesses an input bonding pad within its own area of the integrated circuit. We have built our prototypes for a 4 × 4 microelectrode array. The circuit was fabricated on a 2.2 mm × 2.2 mm chip using the AMIS 1.5 μm process; the circuit level details can be found in [20] although the current version has been slightly modified for better low-noise performance and bias stability. The overall performance characteristics are summarized in Table 1.

**Figure 3.**
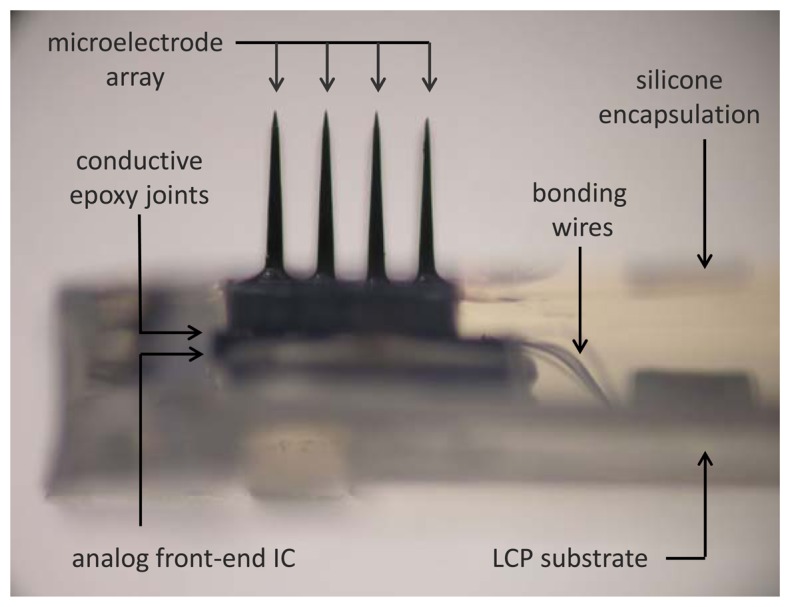
A photographic view of the front-end integrated unit after silicone encapsulation is shown. The height of the microelectrodes serves as a scale bar with a length of 1.5 mm.

As for the chip-to-chip integration of the microelectronic IC with the neural probe array, we designed the amplifier array with a pitch of 400 μm to exactly match the spacing of the extracellular electrodes in the neural probe array. Bonding of each input pad to its corresponding electrode can be performed with a low stress flip-chip bonding technique using a conventional flip chip bonder (model M9, RD Automation, Inc.) with micro-patterned conductive epoxy (H20E, Epoxy Technologies, Inc., Billerica, MA, USA). Figure 3 shows a photographic view of the hybrid integrated unit after silicone encapsulation.

#### 3.1.1. Physical Evaluation of the Neural Sensor Array

After full integration and silicone encapsulation, a cortical neural sensor array was evaluated by bench-top physical testing. The neural sensor was characterized by immersing its probe electrodes into a standard artificial cerebrospinal fluid (ACSF), a physiological saline solution, and applying a periodic “pseudo-spike” (simulated action potential) signal from a function generator through a silver chloride reference electrode. The results verified that our integrated neurosensor is suitable for recording neural activities such as action potentials [21].

#### 3.1.2. *In-Vivo* Testing of the Neural Sensor Array

In the in-vivo neural microsystem evaluation step, we placed an acute implant of the front end of our sensor in the brain of a rat under anesthesia and recorded the neural activity from the somatosensory region of the brain. Male Sprague-Dawley rats were used, and the anesthesia was induced by pentobarbital (initial dose of 80 mg/kg with an additional dose of 30 mg/kg if necessary). During the recording session, the depth of anesthesia was controlled by monitoring the tail-pinch response, corneal reflex, and respiration rate. The monolithic front-end recording device was implanted by a pneumatic impulse inserter, such that the electrode tips reached approximately 1 mm deep from the surface of the brain (near or within layer IV). Fine adjustments of the penetration depth of the microelectrode into the cortex were made by micromanipulator while audio-visually monitoring neural activities. The results were verified by a collateral experiment using a passive single-wire electrode. This *in vivo* animal experiment shows that the CMOS integrated analog front end of the microsystem performs quite satisfactorily as a cortical implant as seen in our previous work [21]. This critical evaluation step is also used for the full system performance characterization, which is described in Section 4.

### 3.2. Flexible Substrate and Dual Panel Design

As outlined above (Figure 1), our implantable microsystem design is based on a dual panel concept, where due to thermal and other considerations the physical layout of the implant separates the microelectrode/CMOS preamplifier assembly and the support components that provide the serial digital data stream output. From our thermal models, our design limits the temperature rise at the cortex to less than 0.5 °C for a full 100-channel system. The common substrate for the entire microsystem is a patterned polyimide (PI) that provides all the internal electrical interconnect wiring between discrete microelectronic chips while maintaining biocompatibility and physical reliability [25,26].

**Figure 4.**
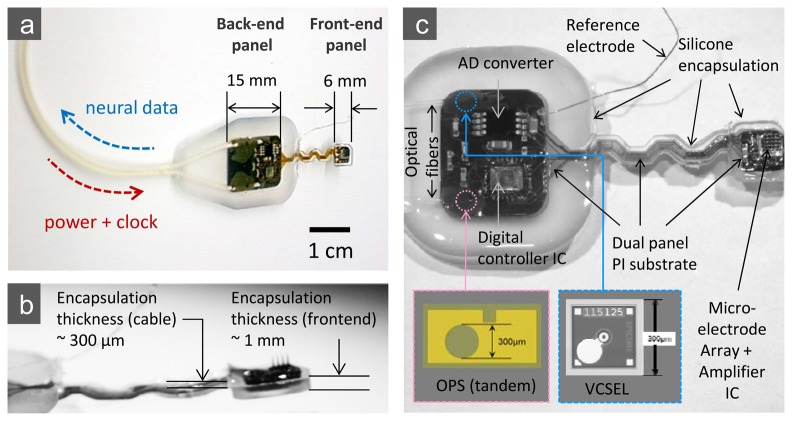
Photograph of the completed microsystem detailing the layout of peripheral microelectronic and (infrared) telemetry components. (**a**) Overall microsystem structure with front- and back-end dimensions; (**b**) side view of the encapsulation profile at the front- and back end connection (serpentine metal patterns and thinner encapsulation for the better flexibility); (**c**) images of the electronic and optoelectronic components in the microsystem—microscope images of the optical power supply (OPS) and VCSEL optoelectronic device components are shown separately at the bottom.

The design provides a flexible, integrated interconnect between the analog front end and the back panel, which houses an ADC, a digital controller chip, and optoelectronic modules, through a set of metal patterns (Cr/Ni/Cu/Ni/Au) on 50 μm-thick polyimide film (see also the photographic image of the completed microsystem in Figure 4). This flexible interconnect allows easy placement of the electrode array and some relative motion between the array and support electronics when implanted.

### 3.3. Design and Implementation of Back End Peripheral Circuitry

As outlined above and illustrated in Figure 4, the back panel of our 16-channel microsystem array platform uses peripheral circuitry and telemetry components on a separate area of the substrate that is electrically connected to the “front end” sensor assembly along the flexible polyimide substrate. This peripheral circuitry (the “back end” of the implant) includes a low power A/D converter, a digital controller chip, a semiconductor microcrystal photovoltaic energy converter, and an infrared data link that employs a low-threshold-current semiconductor laser diode (VCSEL).

#### 3.3.1. Microelectronic Components

The analog-to-digital converter chosen for our 16-channel test system is a low power AD7495BRM ADC from Analog Devices, Inc. (Norwood, MA, USA) that is a 12-bit successive-approximation serial converter capable of 1 Msps (mega samples per second) conversion. Here it is used at 640 Ksps (kilo samples per second) to give a per channel 40 Ksps conversion rate. The converter is in a 1 mm thick, conventional micro-SO8 package. We use a 15.36 MHz clock and allow twenty-four clock cycles per conversion.

All digital control and multiplexing functions are incorporated into a single custom digital controller integrated circuit that directs the electronic traffic for the ADC and amplifier circuits. The controller chip performs four principal functions. First, it generates a full CMOS-level clock signal from a small sinusoidal signal separated from the output of the input power (here the “optical power supply”). Second, it uses a pseudorandom bit generator (PRBG) to create a periodic, 24-bit synchronization code, and it multiplexes that with the output of the ADC converter. This code is detected in the data to mark the beginning of each frame of data. Third, it provides the driver to apply the multiplexed digital signal to drive the laser diode (VCSEL), which is used to transmit the digital optical data out by infrared light guided by an optical fiber or exiting directly through the skin. Finally, the device contains a four-bit counter that supplies the address lines for channel selection in the analog multiplexer on the amplifier integrated circuit. The controller was designed using custom layout for the analog sections for clock generation and VCSEL driver and using standard cells for the digital counters and control. The chip was fabricated with the AMI 0.5-micron process through MOSIS and is integrated onto the full microsystem as shown in Figure 4. Table 2 shows the total power consumption of the microsystem and the breakdown for each component.

#### 3.3.2. Optoelectronics Components

The version of the completed microsystem that we report here acquires its supply power using infrared light coupled by optical fiber to a unique high-efficiency photovoltaic converter, which is the microcrystal on-chip “optical power supply” (OPS) in Figure 4 and is described here in some detail. In this paper, we choose to highlight an optical means of powering the microsystem due to our long-term goal of building an entirely body-implantable system that includes spike decoding and both cortical and muscle stimulation based on computing systems implanted thoracically (e.g., into the chest or abdominal cavity). In this case, the biocompatibility and large bandwidth of an optical fiber threaded from the head through the body are substantial advantages for the entire prosthetic system, including power delivery by the fiber to the brain implantable microsystem. There have been some reports regarding optical power delivery and data transmission for biomedical implants, but none specifically for neural sensing applications [27,28].

**Figure 5.**
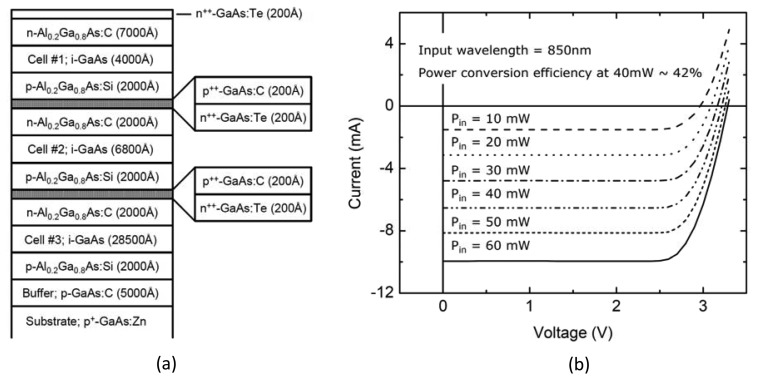
Schematic of the GaAs/AlGaAs heterostructures for a photovoltaic energy converter device (**a**), and current-voltage characteristics of a fully packaged photovoltaic energy converter device under various levels of optical illumination (**b**).

The microcrystal photovoltaic energy converter used to fabricate the OPS components was designed using basic semiconductor device physics principles and computational analyses to create a custom multilayer optoelectronic material. To deliver the requisite voltage, we focused on three vertically stacked semiconductor photovoltaic cells based on crystalline GaAs/AlGaAs heterojunctions. After design and simulation work, the wafers were grown by metallo-organic chemical vapor deposition (MOCVD) by contracting a custom semiconductor epitaxial wafer foundry (IQE, Inc., St. Mellons, Cardiff, UK). Related multi-junction tandem structures have been used recently for high efficiency solar cells and, notably, for space applications [29,30]. The epitaxial wafer material consisted of three p-Al_0.2_Ga_0.8_As/i-GaAs/n-Al_0.2_Ga_0.8_As heterojunctions with optimally designed thicknesses for maximum efficiency that were serially connected via two ultra-thin GaAs p/n tunnel junctions. The detailed layering scheme of the wafer material is shown in Figure 5(a). After delivery, the material was processed into circular aperture devices using standard microelectronic fabrication techniques, with Pd/Ge/Au (100Å/500Å/1,200Å) and Ti/Au (100Å/1,500Å) layers for the n- and p-type ohmic contacts, respectively. A standard multimode silica optical fiber with a 62.5 μm core diameter is aligned to the aperture of a device with a micro-positioning stage, while the light conversion efficiency of the link is actively monitored. The optical fiber is then affixed to the device at peak conversion efficiency with UV curable epoxy (NOA76, Norland Products, Inc., Cranbury, NJ, USA) before being permanently mounted to the silicon sub-mount or directly onto a polyimide substrate using a thermally conductive epoxy (H70E-4, Epoxy Technology, Inc. Billerica, MA, USA). A plan view of a finished device is shown at the bottom of Figure 4, as is its location within the overall “back end” in a full-view photograph of the completed microsystem.

The characteristics of a fully packaged, fiber coupled photovoltaic energy converter device are shown in Figure 5(b). The “optical-to-electrical” power conversion efficiency of the device at 2.8 V (the normal operating voltage of the preamplifier/CMOS IC) is over 40% with an untreated semiconductor-epoxy interface, which accounts for approximately 20% loss that can be easily eliminated by the use of a dielectric anti-reflection coating to extend the efficiency to over 50%.

In the case where incoming fiber-guided IR light provides power for the microsystem of Figure 4, we also simultaneously modulated the external 850 nm semiconductor laser power source to provide the clock signal for the ADC conversion. The rationale for co-delivering the clock signal with the optical power rather than generating it within the implanted digital module is as follows. First, the comparator circuitry to extract the clock signal uses much less power than, e.g., an on-board crystal oscillator. Second, it is not necessary to extract the clock from the return data by means of a phase-locked-loop or similar techniques because the clock originates in the same circuits that decode the return signal. Thus the return data can employ a very simple NRZ coding scheme that is the natural format of both the ADC and the PRBG.

**Figure 6.**
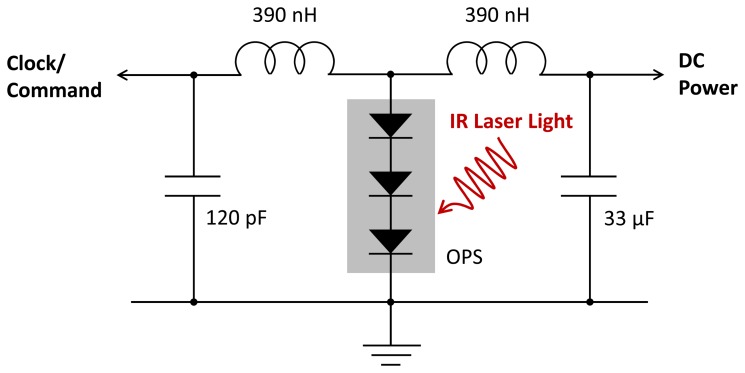
The LC circuit to separate the DC power and the 15.36 MHz sinusoidal clock signal from the output of the optical power supply (OPS).

To implement this scheme, we modulated the current in the laser source diode by a 15.36 MHz square wave at 40 percent of the mean current. Figure 6 shows the LC circuit that separated the output of the microcrystal OPS into a DC signal to power all the circuitry and a sinusoidal signal for the clock comparator in the digital controller. The 0.33 μF capacitor eliminates any RF voltage on that supply. The two coils in series effectively form a parallel resonant circuit with the smaller 120 pF capacitor. The OPS has an equivalent circuit, which is a current source in parallel with a shunt resistor and capacitor. When the shunt impedance is high, the OPS current drives the resonant circuit and produces a 180 mV sinusoidal signal. The clock comparator is a high-gain differential-input amplifier with a low pass feedback network that assures that the master clock signal has an approximately 50 percent duty cycle. However, it is important that the average laser power into the input optical fiber be optimized. If it is too low, the DC voltage falls below the 2.8 V required for correct operation of the ADC and amplifier. If it is too high, the shunt capacitance increases due to excess stored charge in the forward-biased diodes while the shunt resistance drops as the device approaches open-circuit conditions. The low shunt impedance then reduces the sinusoidal signal below the threshold for reliable comparator operation

### 3.4. Encapsulation

In contrast with commonly employed “passive” cortical microelectrode probes which are cabled percutaneously to external electronics, implanted active microelectronic components present significant encapsulation challenges. The problem of encapsulating electronic devices for chronic cortical neural recording and stimulation poses several unique challenges not faced by devices located elsewhere in the body; in particular, the requirements for low thermal dissipation and a very thin package. Functionally the encapsulation serves three purposes: to protect the body from possible non-biocompatible materials, to protect the electronic devices from body fluids, and to protect the assembly from mechanical stress. The key issue is to prevent the transport of water or ions dissolvable in water from penetrating the encapsulant in either direction.

The maximum thickness of our microsystem assembly is on the order of 1–2 mm and is limited by the space between the dura and the skull where the “back end” panel is inserted. Work by our clinical collaborators indicates that this is an important and perhaps unappreciated variable because tall implants sink into the cortex in a manner that is still poorly understood. Based on the positional stability we now observe, we believe there is an upper bound on the amount of encapsulant material that may be placed between current carrying conductors (e.g., bond wires or printed circuit traces), which is of the order of 1 mm. Multiple, closely spaced pathways demand an encapsulation system that is statistically reliable and thin enough not to significantly alter the electrode geometry; our approach, in contrast to others, has been guided by this principle.

In this phase of our development work on cortical microsystems, we have concentrated on developing encapsulation procedures using a specific silicone polymer (R-2188, NuSil Technology LLC, Carpinteria, CA, USA) to encase the assembled circuits. This encapsulation material system is known to produce satisfactory chronic packages but has been associated with variable reliability, which is still poorly understood [31]. Our encapsulation process begins with the preparation of the assembled microsystem by ultrasonic cleaning, and the unit is baked at 110 °C for 5 minutes. After all the components on the substrate are carefully under-filled with silicone, the mold cavity is filled by slowly dripping silicone into one corner at each end of the mold. Finally, the silicone is cured by heating the mold to 120 °C and holding that temperature for 4 hours. To reduce the wicking of silicone up the electrodes during curing, a jet of dry nitrogen is blown on that region. We also note that the thickness requirements for the encapsulation of different parts of the microsystem vary; the front-end unit requires thinner encapsulation than that for the back-end due to limited volume in the sub-cranial space as described above, and the connection cable between the front- and the back-end requires the thinnest encapsulation for the maximum flexibility. We employed a multi-level mold structure to make a thick (∼2 mm for the back-end), a thinner (∼1 mm for the front-end), and the thinnest (∼0.3 mm for the connection bridge) silicone profiles in a single encapsulation procedure. Details of the encapsulation structure are shown in the photographic images of Figure 4.

## 4. Performance of Full Microsystem

In this section we summarize the performance of the 16-channel fully integrated microsystem in bench top feasibility studies. Although a single optical fiber can serve both as the input path for incoming power and clock signals and as the output for the digitized optical data stream, we are currently still optimizing the on-chip optical alignment details of the fiber while adding miniaturized fiber couplers and beam splitters for optical access to the OPS and VCSEL microcrystals, respectively. To evaluate the salient features of the microsystem, we chose to separate the input and output optical paths by employing two standard multimode silica optical fibers with diameters of 62.5 μm. One fiber delivered the power and clock signals (the up-stream link for power delivery and forward telemetry), and the other fiber delivered the neural data transmission (the down-stream link for backward telemetry). The up-stream fiber optic link was connected to a compact fiber coupled diode laser (SRT-F850S-60, Micro Laser Systems, Inc., Garden Grove, CA, USA) with an external modulator, which delivered a 15.36 MHz intensity-modulated light signal with an average power of 40 mW at a wavelength of 850 nm to the OPS photovoltaic energy converter on the back-panel of the microsystem. The down-stream fiber optic link was connected to a miniature fiber optic photoreceiver (HFBR-2416, Agilent Technologies, Inc., Santa Clara, CA, USA), which converted the pulse-coded modulated light signal into a digital stream of electrical pulses (a TTL signal) for real-time reconstruction and storage of the neural-type signal recordings in an IBM-compatible PC though a semi-custom designed digital interface board.

**Figure 7.**
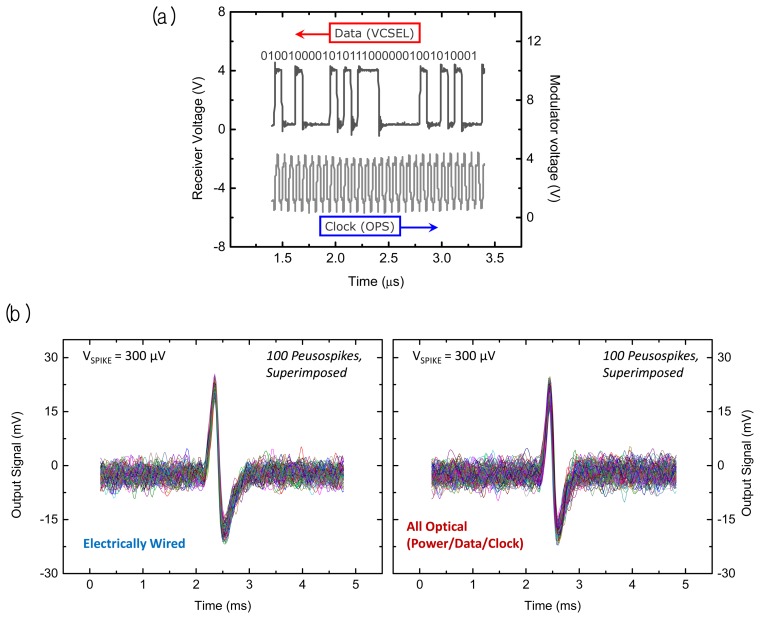
Bench top testing of the all-optical neural sensing microsystem; (**a**) optical digital data segment from a portion of a spike event (top) and the corresponding clock sampling trace (bottom); (**b**) comparison of system performance using a pseudospike recording test in a saline bath. A 300 μV bipolar spike signal is measured by the all-optical telemetry/power system (right panel) and a system with electrically wired telemetry and power links (left panel).

A performance demonstration of the full 16 channel microsystem is shown in Figure 7(a). The upper trace in that figure displays a portion of a digitized optical data output measured by a photoreceiver that was spatially separated from the microsystem in a benchtop experiment. Here the microelectrode array has been excited by a series of ‘pseudospikes’ by applying a bipolar transient voltage through a wire to a saline (ACSF) solution in which the microsystem unit was immersed. Note the microsecond timescale in the upper data stream traceas opposed to the digital sampling rate indicated in the bottom clock trace. The pulse-coded modulation (PCM) data at the photoreceiver demonstrates a robust fiber optic data link in the present microsystem. The lower trace originates from the sinusoidal waveform applied to the external diode laser power source, which is current-modulated to generate an infrared optical input with a 15.36 MHz clock signal.

After reconstruction of the received optical data stream to an analog form, we can compare the pseudospike signals from an electrically wired system to the signals from the present all-optical microsystem as shown in Figure 7(b). The left panel is acquired by electrically accessing the front end of the system with a set of wires under specific test conditions (*i.e.*, a train of bipolar pseudospikes applied to the ACSF bath with a peak-to-peak amplitude of 300 μV at 50 Hz). The signals acquired from the all-optical system on the right panel show almost the same preamplifier gain of 42 dB as the front end microelectronic system, with comparable input noise characteristics.

**Figure 8.**
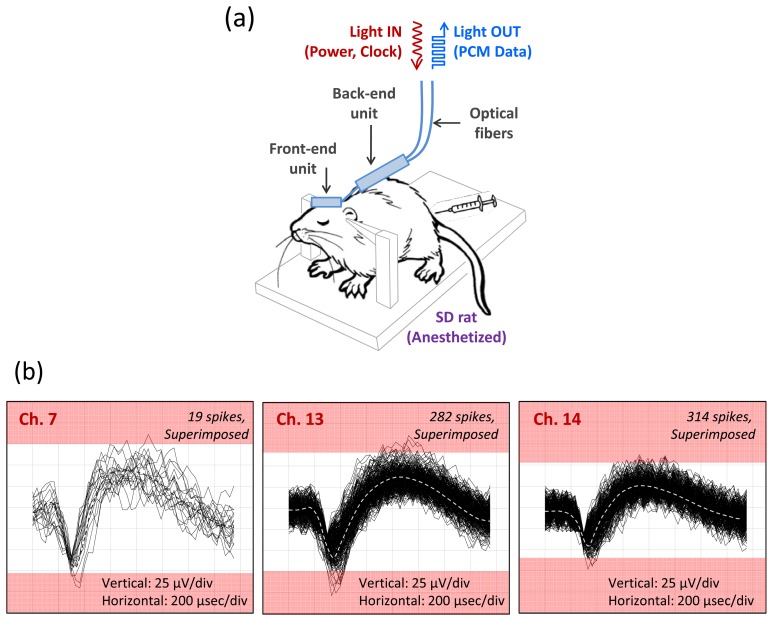
*In vivo* recording of neural activity from the somatosensory cortex of a rat made by the fully integrated 16 channel neural sensing microsystem equipped with fiber-optic data transmission and power delivery components. (**a**) Schematic illustration of *in vivo* recording setup with anesthetized rat; (**b**) An example of the neural signals showing three active channels simultaneously recorded with the microsystem, where the spikes (single unit activities) are superimposed with an average value shown by a dotted line as a guide to the eye.

After validating the system performance in a bench top test, we evaluated the fully integrated microsystem in a real biological environment using rodents as described in Section 3.1.1. Due to anatomical size constraints, we implanted the analog front end of our microsystem in the somatosensory cortex of anesthetized rats and recorded well-characterized spontaneous neural activity and evoked neural activity from sensory stimulation of the whiskers and posterior skin. The recorded neural activity is correlated well with the sensory stimuli and shows a high rate of burst activity. A typical *in-vivo* recording of the neural activity from an anesthetized rat is shown in Figure 8. Only three channels are shown active, and we attribute the relatively low channel yield to either suppression of the neural activities by the anesthetizing agent (pentobarbital or ketamine/xylazine) and/or a mismatch between the recording plane (defined by the tips of the microelectrode array) and the cortical layer of a rat. Although a continuous effort is being made to improve the channel yield *in vivo* rat experiments, these issues may not be critical to the actual neuroprosthetic applications, considering anatomical and physiological difference between rats and primates.

The result still demonstrates the practical utility of our infrared optical telemetry with an optical power delivery scheme for advanced brain implantable neural sensing microsystems.

## 5. Conclusions

In this paper, we have described our progress in the development of an advanced brain implantable microsystem for the neuromotor prosthesis that includes new optical technologies for a broad bandwidth infrared optical link for (potentially bidirectional) neural data transmission, and a photovoltaic microcrystal energy converter for efficient power delivery to the brain implantable active electronics.

The concept of an optoelectronic power and data link enables considerable system flexibility for future neural prosthesis applications. Extraction of the digitized IR neural signals from the neural interface unit can be performed either via a free space beam from the laser emerging directly through the skin, which is sufficiently transparent in the near infrared, or via a fiber optic strand threaded subcutaneously to other information linking sites in the body (e.g., a thoracic unit). We also note that our photovoltaic energy converter device (or optical power supply) could work as a forward telemetry link by modulating IR input to the device. We have applied only clock signals in our present microsystem, but we could employ command signals on top of the clock signals if amplitude modulation is used to transmit additional information besides the clock frequency; this would be an interesting subject for the future development of an implantable neural sensing microsystem.

Although more conventional metal wiring would provide a simple and efficient solution for the power delivery and would allow a moderate data rate telemetry, our all optical approach could offer MRI-compatible, EMI-free, high data-rate subcutaneous wiring with biocompatible and flexible materials (note that polymeric optical fiber could replace the current version of silica fiber for more flexibility). An additional advantage is that the light transmission is less sensitive to small leakage of body fluid as the electrical conduction in metals is much more vulnerable to ionic fluids. In many circumstances, this would become a critical advantage of optical wiring schemes over simpler metal wiring solutions in totally implantable biomedical device applications. Other optical technologies, such as wavelength division multiplexing, could also be used for a more complex multi-unit neural sensor microsystem network in the future.

We also introduced a dual panel design concept with flexible substrates made of the patterned polyimide, to minimize the risk of thermal damage of the cortical tissues and to facilitate surgical implantation into the brain. While a proof-of-concept, the 16 channel version of this prototype microsystem with both an infrared data transmission link and an optical power delivery scheme has proven to have practical utility as our new platform for a high-performance brain-computer/machine neural interface, some challenges remain for emerging human applications such as long term reliability of the encapsulation, development of the surgical procedure, and system management strategies. These concerns will be addressed more effectively with our on-going efforts in system design, failure mode analysis, and the development of a chronic animal model using non-human primates.

## Figures and Tables

**Table 1.** Performance Characteristics of the Amplifier IC.

**Parameter**	**Simulation**	**Measured**
Noise (RTI 300 Hz to 20 kHz)	3.9 μV_RMS_	4.5 μV_RMS_
High frequency cutoff (−3 dB)	7.5 kHz	7.3 kHz
Low frequency cutoff (−3 dB)	NA	2.5 Hz
Power per amplifier	50 μW	52 μW
Power	1.3 mW	1.3 mW

**Table 2.** Breakdown of Power Dissipation in the Microsystem.

**Component**	**Power Consumption**
**Preamplifier (Total)**	1.3 mW
**Digital Controller**	5 mW
**ADC (AD7495)**	4.5 mW
**Laser (850nm VCSEL)**	2 mW

**Total**	12.8 mW
